# Crystal structure and Hirshfeld surface analysis of *N*′-[(1*E*)-1-(3-oxo-3,4-di­hydro-2*H*-1,4-benzoxazin-6-yl)ethyl­idene]benzohydrazide monohydrate

**DOI:** 10.1107/S2056989025007819

**Published:** 2025-09-05

**Authors:** Sekar Janarthanan, Ganesan Meenambigai, Velayutham Mahalakshmi, Manivel Kavitha, Rajendran Arivu Selvan, Srinivasan Pazhamalai, Sivashanmugam Selvanayagam

**Affiliations:** a Department of Chemistry Annamalai University, Annamalainagar Chidambaram 608 002 India; b PG & Research Department of Physics, Government Arts College, Melur 625 106, India; Vienna University of Technology, Austria

**Keywords:** hydrazone derivatives, inter­molecular hydrogen bonds, Hirshfeld surface analysis, crystal structure

## Abstract

The title compound, C_17_H_15_N_3_O_3_·H_2_O, a hydrazone derivative, crystallizes with one mol­ecule of water. The morpholine ring adopts a twist-boat conformation. Inter­molecular N—H⋯O, O—H⋯O and C—H⋯O hydrogen bonds are responsible for the consolidation of the crystal packing. The inter­molecular inter­actions were qu­anti­fied using Hirshfeld surface analysis, revealing that H⋯H inter­actions contribute most (40.6%) to the crystal packing.

## Abstract 2

The title hydrazone derivative crystallizes with one mol­ecule of water. Inter­molecular N—H⋯O, O—H⋯O and C—H⋯O hydrogen bonds are responsible for the consolidation of the crystal packing.

## 1. Chemical context

Hydrazones, characterized by the —HN—N=C— linkage, are an important class of organic compounds with wide-ranging applications. They are employed as catalysts, bioactive mol­ecules, organic dyes, and liquid crystals, as well as in agriculture as insecticides, sterilants, and herbicides (Meenatchi *et al.*, 2021 ▸; Costa *et al.*, 2025 ▸; Fuh *et al.*, 2012 ▸). Their ability to form hydrogen bonds and coordinate to metal ions enhances their versatility, making them valuable scaffolds in drug design (Karthiga *et al.*, 2025 ▸). Hydrazone derivatives also display a wide range of pharmacological activities, including anti­microbial, anti­cancer, anti­malarial, anti­convulsant, and cardioprotective effects, with several already in clinical use. Examples include isoniazid, an essential anti­tubercular drug, and related analogs such as isocarboxazid, iproniazid, furazolidone, nifuroxazide, nitro­furan­toin, and nitro­furazone, which are employed against various diseases. These cases highlight the therapeutic importance of hydrazide/hydrazone derivatives and their relevance in drug discovery (Teneva *et al.*, 2023 ▸).
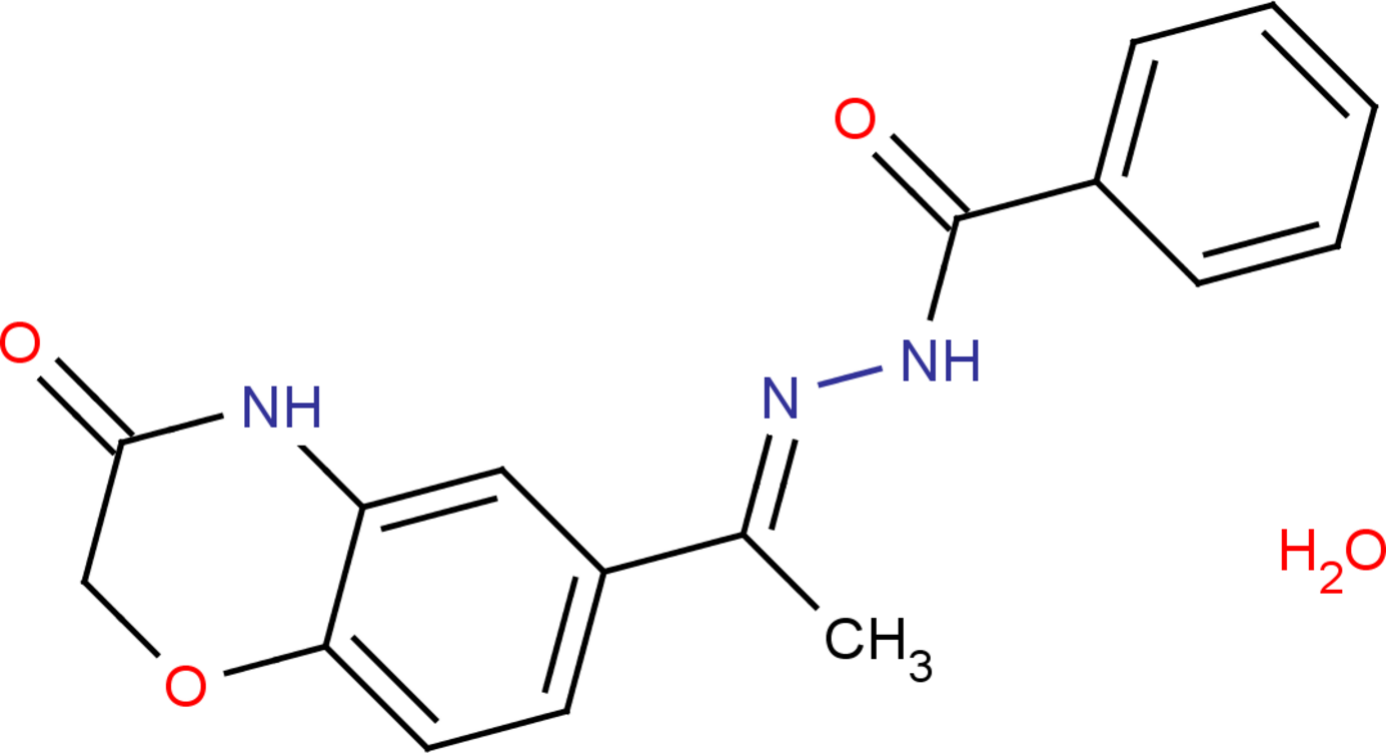


In the context given above, we synthesized a new hydrazone derivative, *N*′-[(1*E*)-1-(3-oxo-3,4-di­hydro-2*H*-1,4-benzoxazin-6-yl)ethyl­idene]benzohydrazide monohydrate, (I), and report here its mol­ecular and crystal structure, and the results of a Hirshfeld surface analysis.

## 2. Structural commentary

**Figure 1:**
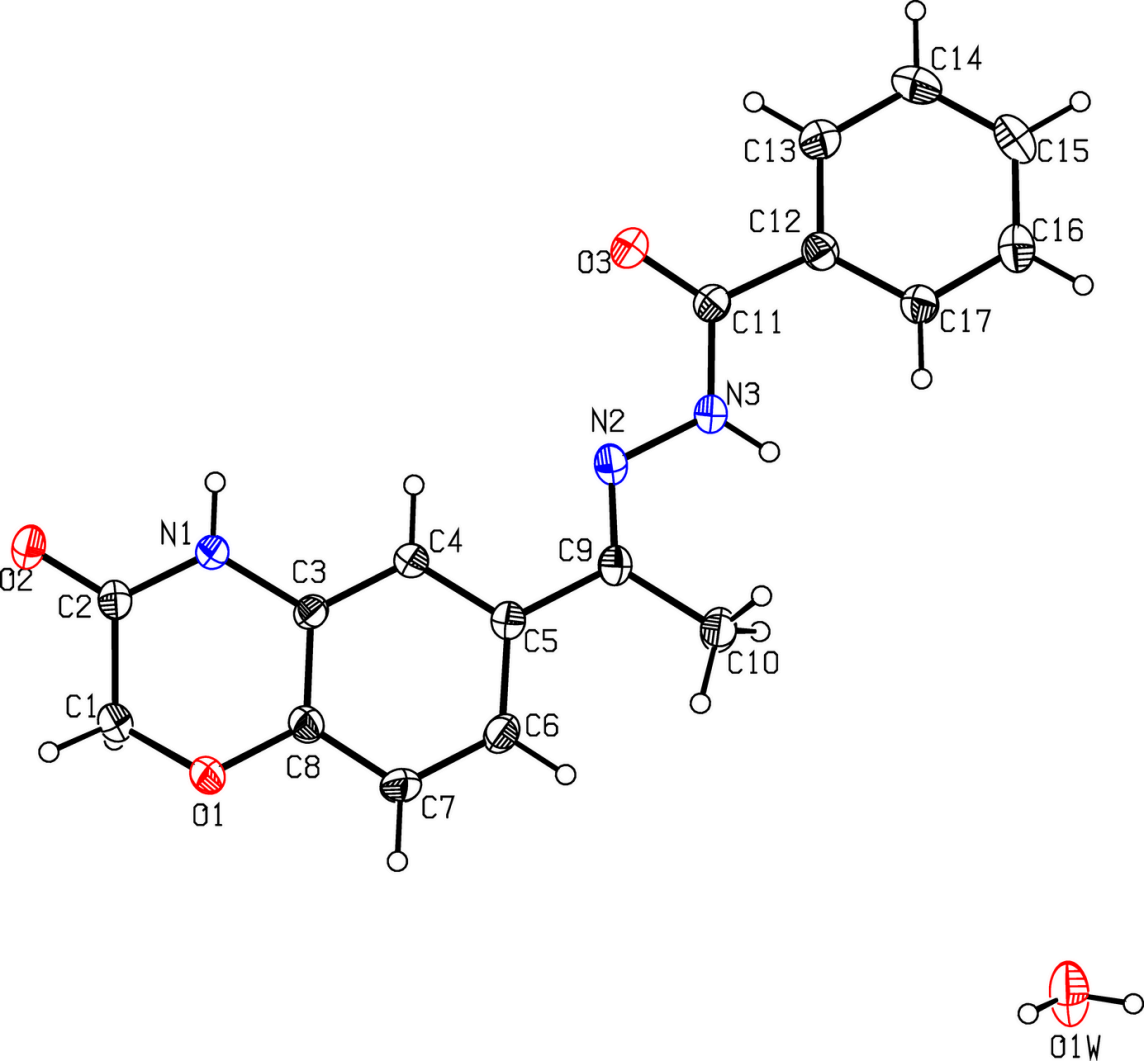
A view of the mol­ecular structure of (I), showing the atom labelling. Displacement ellipsoids are drawn at the 30% probability level.

The mol­ecular structure of (I) is displayed in Fig. 1 ▸. The O2—C2 [1.235 (3) Å], C9—N2 [1.285 (3) Å] and O3—C11 [1.228 (3) Å] bond lengths confirm double-bond character. The morpholine ring adopts a twist-boat conformation with puckering parameters (Cremer & Pople, 1975 ▸) *q*_2_ = 0.322 (2) Å, *q*_3_ = 0.141 (2) Å, *Q*_T_ = 0.352 (2) Å and φ = 30.8 (3)°. Atom C1 deviates by 0.460 (3) Å from the least-squares plane through the remaining five atoms (O1/C2/N1/C3/C8) of the morpholine ring. The mean-plane calculation of the *N*′-[(1*Z*)-ethyl­iden]formohydrazide moiety (C9–C10/N2/N3/C11/O3) reveals that the atoms C10 and O3 deviate by 0.1836 (12) and 2.081 (14) Å, respectively, from the plane. This moiety makes a dihedral angle of 2.81 (13)° with respect to the phenyl ring (C12–C17). This phenyl ring and the phenyl ring (C3–C8) fused to the morpholine ring are oriented at a dihedral angle of 5.67 (10)°.

## 3. Supra­molecular features

**Figure 2:**
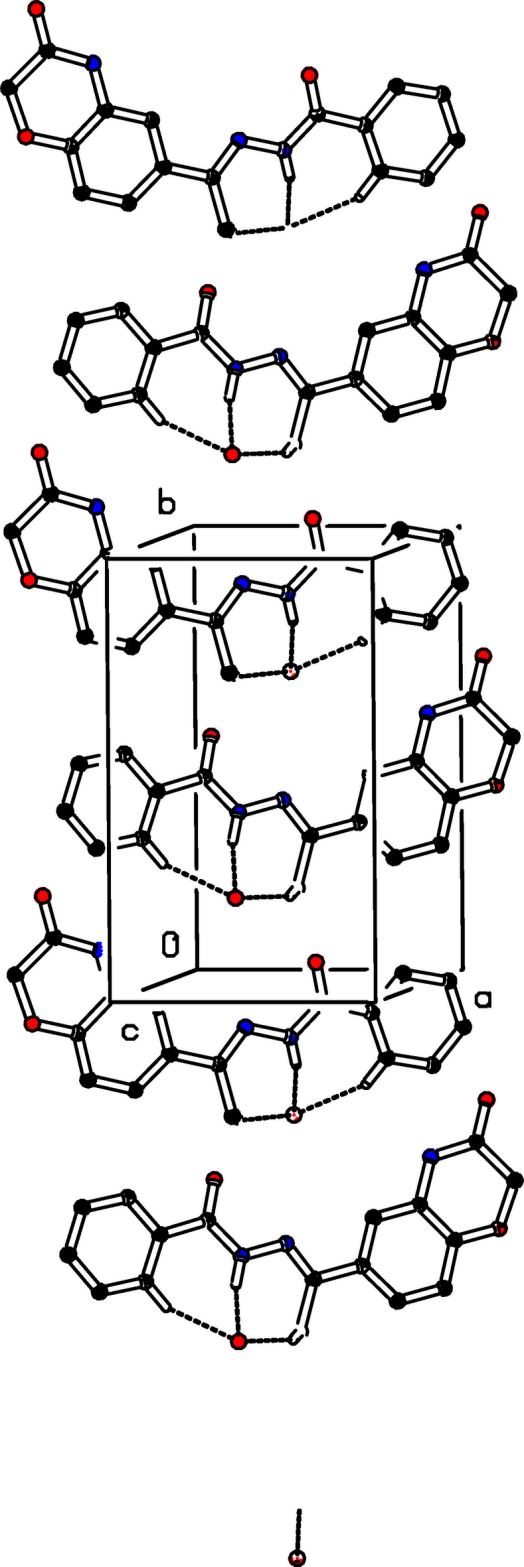
The crystal packing of (I) with N—H⋯O and C—H⋯O hydrogen bonds to the solvent water mol­ecule as an acceptor shown as dashed lines. For clarity, H atoms not involved in these hydrogen bonds have been omitted.

**Figure 3:**
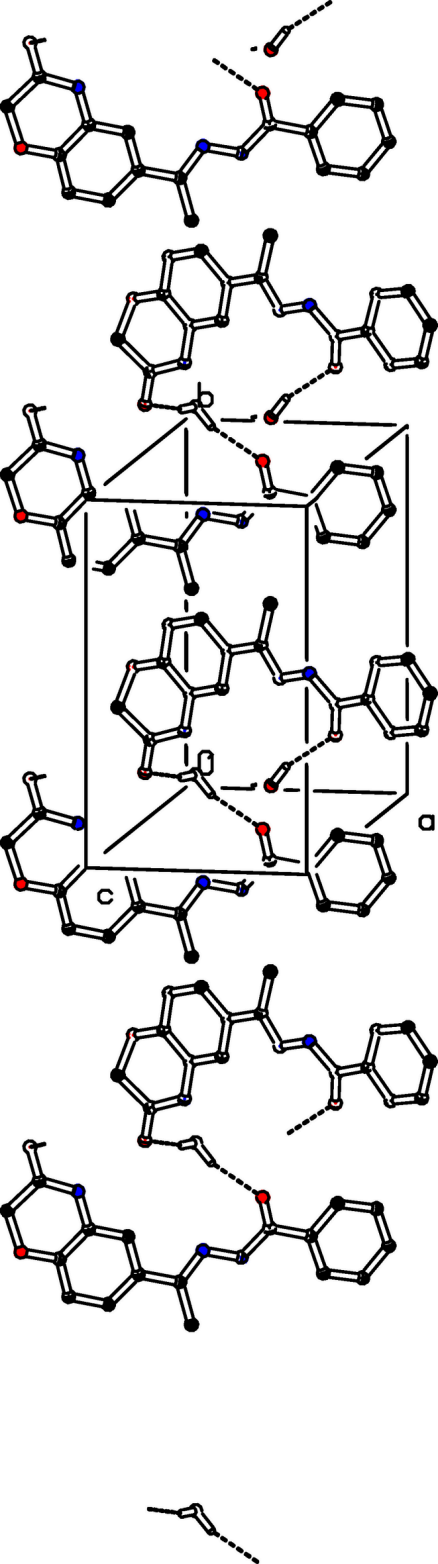
The crystal packing of (I) with O—H⋯O hydrogen bonds involving the water solvent mol­ecule as a donor shown as dashed lines. For clarity, H atoms not involved in these hydrogen bonds have been omitted.

**Figure 4:**
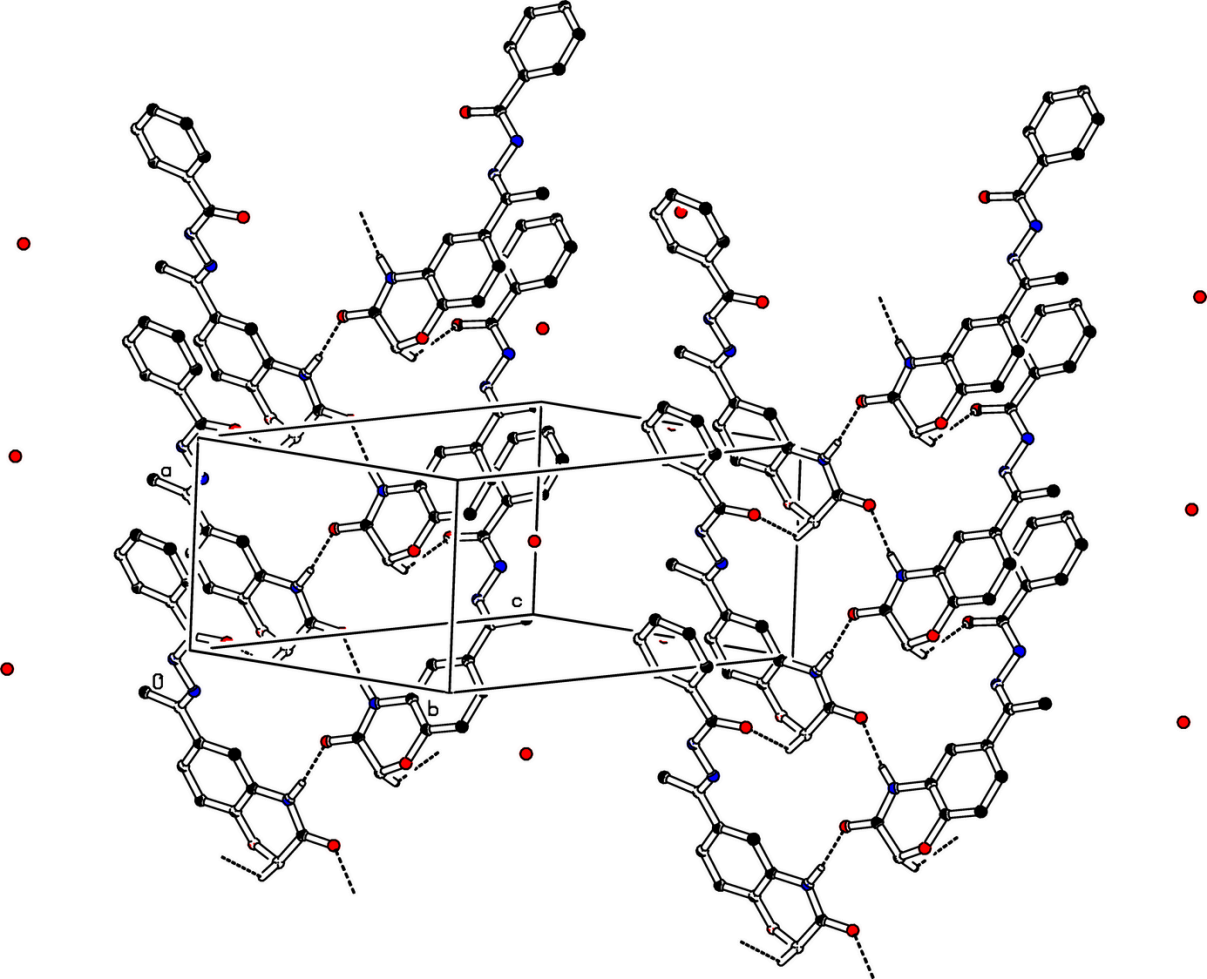
The crystal packing of (I) with N—H⋯O and C—H⋯O hydrogen bonds shown as dashed lines. For clarity, H atoms not involved in these hydrogen bonds have been omitted.

In the crystal of (I), the oxygen atom (O1*W*) of the water mol­ecule plays a major role in the crystal packing, acting both as a donor and an acceptor group in inter­molecular O—H⋯O, N—H⋯O, and C—H⋯O hydrogen bonds (Table 1 ▸). The water mol­ecule O1*W* acts as a trifold acceptor for two C—H⋯O (C10—H10*B*⋯O1*W*^ii^ and C17—H17⋯O1*W*^ii^) and one N3—H3⋯O1*W*^ii^ hydrogen bond (Fig. 2 ▸). It is a donor for two O—H⋯O hydrogen bonds (O1*W*—H1*W*⋯O2^v^ and O1*W*—H2*W*⋯O3^vi^; Fig. 3 ▸). Mol­ecules associate further into *C*(4) chains by N1—H1⋯O2^i^ hydrogen bonds running parallel to [100]. In addition, C16—H16⋯O3^iv^ hydrogen bonds form *C*(6) chains running parallel to [110] (Fig. 4 ▸).

## 4. Hirshfeld surface analysis

**Figure 5:**
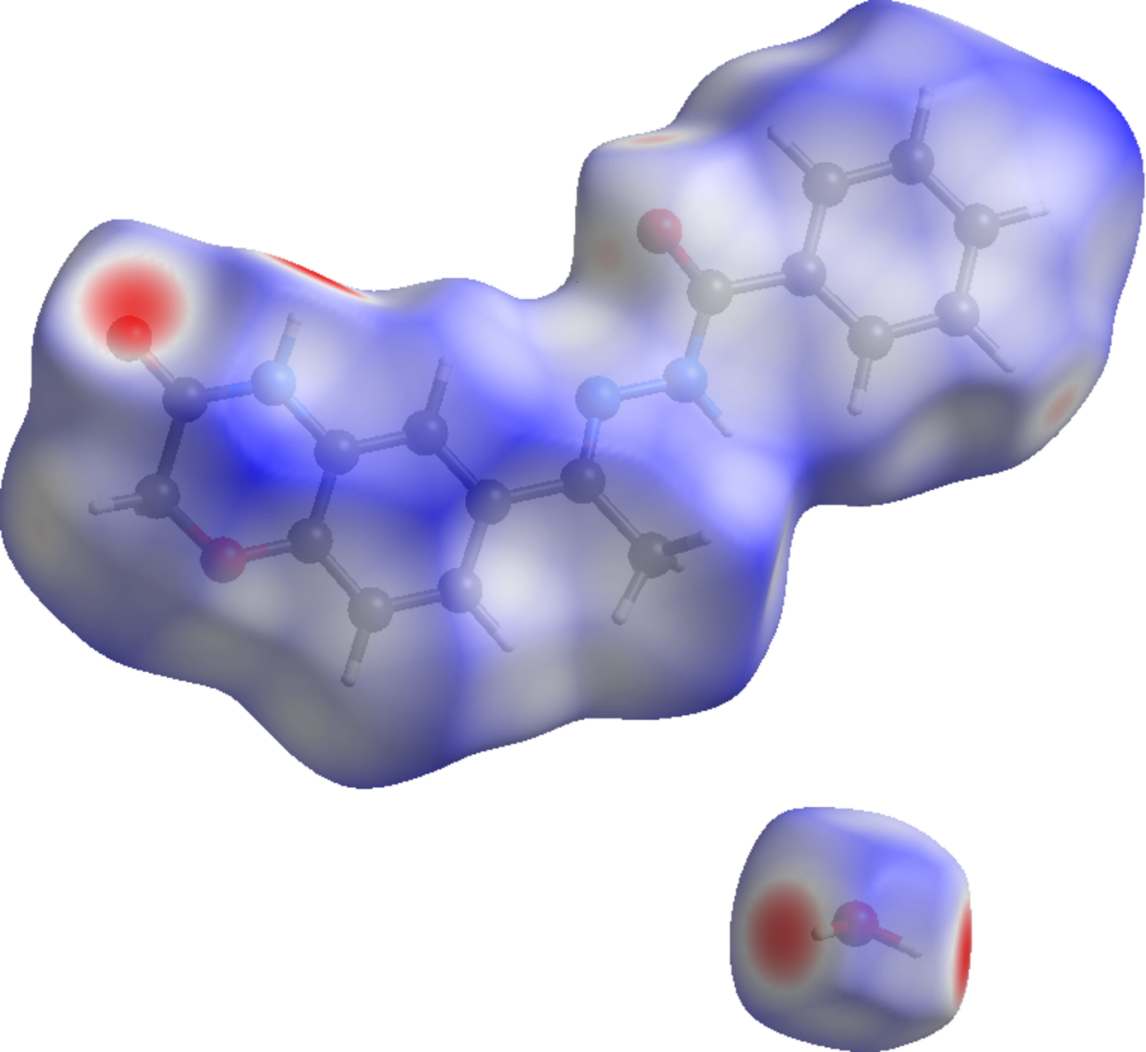
A view of the Hirshfeld surface mapped over *d*_norm_ for compound (I).

To further characterize and qu­antify the inter­molecular inter­actions in the title compound, a Hirshfeld surface (HS) analysis (Spackman & Jayatilaka, 2009 ▸) was carried out using *CrystalExplorer* (Spackman *et al.*, 2021 ▸). The HS mapped over *d*_norm_ is illustrated in Fig. 5 ▸ where the deep-red spots at O2, O3, O1*W* and H1 are indicative of the inter­molecular N—H⋯O, O—H⋯O and C—H⋯O hydrogen bonds discussed above.

**Figure 6:**
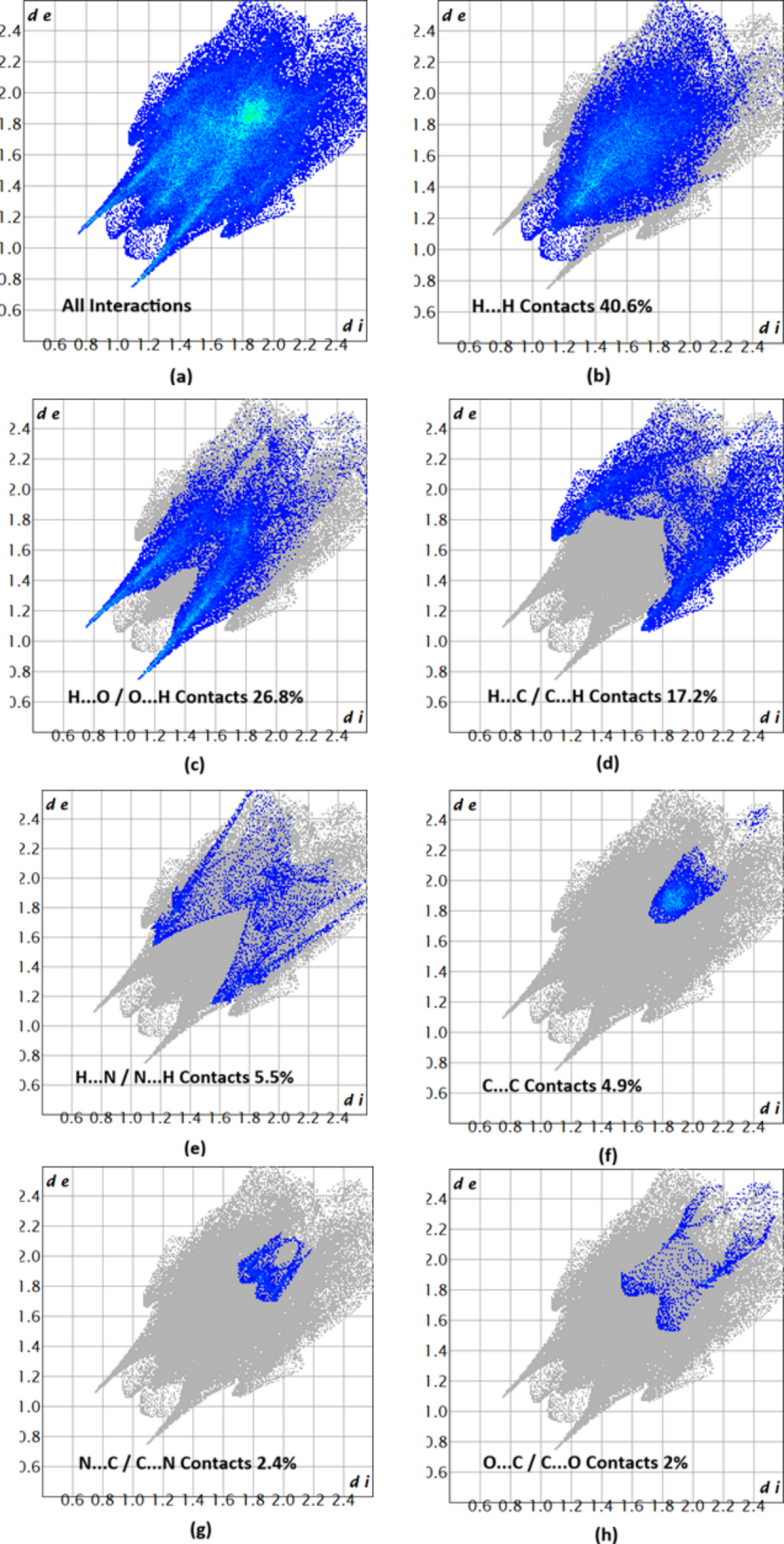
Two-dimensional fingerprint plots for (I), showing (*a*) all inter­actions, and delineated into (*b*) H⋯H, (*c*) H⋯O/O⋯H, (*d*) H⋯C/C⋯H, (*e*) H⋯N/N⋯H (*f*)C⋯C, (*g*) N⋯C/C⋯N and (*h*) O⋯C/C⋯O inter­actions with the corresponding percentages contribution. The *d*_i_ and *d*_e_ values are the closest inter­nal and external distances (in Å) from given points on the Hirshfeld surface.

The associated two-dimensional fingerprint plots (McKinnon *et al.*, 2007 ▸) are displayed in Fig. 6 ▸. They provide qu­anti­tative information about the non-covalent inter­actions in the crystal packing in terms of the percentage contribution of the inter­atomic contacts (Spackman & McKinnon, 2002 ▸). The HS analysis revealed that H⋯H and H⋯O/O⋯H contacts are the main contributors to the crystal packing, followed by H⋯C/C⋯H, H⋯N/N⋯H, C⋯C, N⋯C/C⋯N and O⋯C/C⋯O contacts.

## 5. Synthesis and crystallization

A mixture of 4-benzohydrazide (2 mmol) and 6-acetyl-2*H*-benzo[*b*][1,4]oxazin-3(4*H*)-one (2 mmol) was dissolved in methanol (25 ml) containing a few drops of glacial acetic acid to obtain a clear solution. The reaction mixture was transferred to a round-bottom flask and refluxed for 3 h with continuous stirring. The progress of the reaction was monitored by thin-layer chromatography (TLC). Upon completion, the solvent was removed under reduced pressure, affording a solid residue. The crude product was collected, washed with cold methanol to remove impurities, and subsequently recrystallized from hot methanol to yield a pure product of (I).

## 6. Refinement

Crystal data, data collection and structure refinement details are summarized in Table 2 ▸. Atoms H1*W* and H2*W* were located in a difference-Fourier map and freely refined. Other H atoms were placed in idealized positions and allowed to ride on their parent atoms with N—H = 0.86 Å and C—H = 0.93–0.97 Å, and with *U*_iso_(H) = 1.5*U*_eq_(C-meth­yl) and 1.2*U*_eq_(C or N) for other H atoms.

## Supplementary Material

Crystal structure: contains datablock(s) I, global. DOI: 10.1107/S2056989025007819/wm5768sup1.cif

Structure factors: contains datablock(s) I. DOI: 10.1107/S2056989025007819/wm5768Isup2.hkl

Supporting information file. DOI: 10.1107/S2056989025007819/wm5768Isup3.cml

CCDC reference: 2480218

Additional supporting information:  crystallographic information; 3D view; checkCIF report

## Figures and Tables

**Table 1:** Hydrogen-bond geometry (Å, °)

*D*—H⋯*A*	*D*—H	H⋯*A*	*D*⋯*A*	*D*—H⋯*A*
N1—H1⋯O2^i^	0.86	1.99	2.847 (3)	173
N3—H3⋯O1*W*^ii^	0.86	2.25	3.037 (3)	152
O1*W*—H1*W*⋯O2^iii^	0.83 (1)	2.10 (2)	2.925 (3)	169 (5)
O1*W*—H2*W*⋯O3^iv^	0.83 (1)	2.05 (2)	2.847 (3)	162 (3)
C10—H10*B*⋯O1*W*^ii^	0.96	2.34	3.200 (3)	150
C17—H17⋯O1*W*^ii^	0.93	2.52	3.228 (3)	133
C1—H1*B*⋯O3^v^	0.97	2.57	3.286 (3)	131
C16—H16⋯O3^vi^	0.93	2.58	3.447 (3)	155

Symmetry codes: (i) 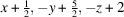; (ii) 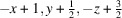; (iii) 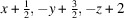; (iv) 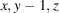; (v) 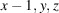; (vi) 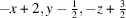.

**Table 2:** Experimental details

Crystal data	
Chemical formula	C_17_H_15_N_3_O_3_·H_2_O
*M*_r_	327.33
Crystal system, space group	Orthorhombic, *P*2_1_2_1_2_1_
Temperature (K)	299
*a*, *b*, *c* (Å)	7.4104 (3), 12.2781 (5), 17.3257 (7)
*V* (Å^3^)	1576.39 (11)
*Z*	4
Radiation type	Mo *K*α
μ (mm^−1^)	0.10
Crystal size (mm)	0.27 × 0.13 × 0.12

Data collection	
Diffractometer	Bruker APEXII CCD
Absorption correction	Multi-scan (*SADABS*; Krause *et al.*, 2015 ▸)
*T*_min_, *T*_max_	0.974, 0.988
No. of measured, independent and observed [*I* > 2σ(*I*)] reflections	15825, 3894, 2868
*R*_int_	0.036
(sin θ/λ)_max_ (Å^−1^)	0.667

Refinement	
*R*[*F*^2^ > 2σ(*F*^2^)], *wR*(*F*^2^), *S*	0.044, 0.111, 1.05
No. of reflections	3894
No. of parameters	226
No. of restraints	2
H-atom treatment	H atoms treated by a mixture of independent and constrained refinement
Δρ_max_, Δρ_min_ (e Å^−3^)	0.19, −0.25
Absolute structure	Refined as an inversion twin
Absolute structure parameter	−0.4 (15)

Computer programs: *APEX3* and *SAINT* (Bruker, 2017 ▸), *SHELXT* (Sheldrick, 2015*a* ▸), *ORTEP-3 for Windows* (Farrugia, 2012 ▸), *SHELXL* (Sheldrick, 2015*b* ▸) and *PLATON* (Spek, 2020 ▸).

## supplementary crystallographic information

### N'-[(1E)-1-(3-Oxo-3,4-dihydro-2H-1,4-benzoxazin-6-yl)ethylidene]benzohydrazide monohydrate Crystal data

**Table:**

C_17_H_15_N_3_O_3_·H_2_O	*D*_x_ = 1.379 Mg m^−^^3^
*M**_r_* = 327.33	Mo *K*α radiation, λ = 0.71073 Å
Orthorhombic, *P*2_1_2_1_2_1_	Cell parameters from 5917 reflections
*a* = 7.4104 (3) Å	θ = 3.0–23.2°
*b* = 12.2781 (5) Å	µ = 0.10 mm^−^^1^
*c* = 17.3257 (7) Å	*T* = 299 K
*V* = 1576.39 (11) Å^3^	Block, colourless
*Z* = 4	0.27 × 0.13 × 0.12 mm
*F*(000) = 688	

### N'-[(1E)-1-(3-Oxo-3,4-dihydro-2H-1,4-benzoxazin-6-yl)ethylidene]benzohydrazide monohydrate Data collection

**Table:**

Bruker APEXII CCD diffractometer	2868 reflections with *I* > 2σ(*I*)
Radiation source: i-mu-s microfocus source	*R*_int_ = 0.036
ω and φ scans	θ_max_ = 28.3°, θ_min_ = 2.0°
Absorption correction: multi-scan (SADABS; Krause *et al.*, 2015)	*h* = −9→7
*T*_min_ = 0.974, *T*_max_ = 0.988	*k* = −16→16
15825 measured reflections	*l* = −23→22
3894 independent reflections	

### N'-[(1E)-1-(3-Oxo-3,4-dihydro-2H-1,4-benzoxazin-6-yl)ethylidene]benzohydrazide monohydrate Refinement

**Table:**

Refinement on *F*^2^	Hydrogen site location: mixed
Least-squares matrix: full	H atoms treated by a mixture of independent and constrained refinement
*R*[*F*^2^ > 2σ(*F*^2^)] = 0.044	*w* = 1/[σ^2^(*F*_o_^2^) + (0.0632*P*)^2^] where *P* = (*F*_o_^2^ + 2*F*_c_^2^)/3
*wR*(*F*^2^) = 0.111	(Δ/σ)_max_ < 0.001
*S* = 1.05	Δρ_max_ = 0.19 e Å^−^^3^
3894 reflections	Δρ_min_ = −0.25 e Å^−^^3^
226 parameters	Absolute structure: Refined as an inversion twin
2 restraints	Absolute structure parameter: −0.4 (15)

### N'-[(1E)-1-(3-Oxo-3,4-dihydro-2H-1,4-benzoxazin-6-yl)ethylidene]benzohydrazide monohydrate Special details

**Table:**

Geometry. All esds (except the esd in the dihedral angle between two l.s. planes) are estimated using the full covariance matrix. The cell esds are taken into account individually in the estimation of esds in distances, angles and torsion angles; correlations between esds in cell parameters are only used when they are defined by crystal symmetry. An approximate (isotropic) treatment of cell esds is used for estimating esds involving l.s. planes.
Refinement. Refined as a 2-component inversion twin.

### N'-[(1E)-1-(3-Oxo-3,4-dihydro-2H-1,4-benzoxazin-6-yl)ethylidene]benzohydrazide monohydrate Fractional atomic coordinates and isotropic or equivalent isotropic displacement parameters (Å^2^)

**Table:**

	*x*	*y*	*z*	*U*_iso_*/*U*_eq_	
H1W	0.377 (7)	0.224 (4)	0.8703 (13)	0.17 (2)*	
H2W	0.486 (4)	0.1690 (19)	0.822 (2)	0.080 (11)*	
O1	−0.2896 (2)	0.95271 (12)	1.00996 (10)	0.0477 (5)	
O2	−0.2428 (2)	1.24134 (13)	1.02249 (10)	0.0469 (5)	
O3	0.7149 (2)	1.07637 (13)	0.81266 (10)	0.0495 (5)	
N1	−0.0451 (3)	1.11560 (15)	0.98123 (11)	0.0371 (5)	
H1	0.041324	1.162300	0.982514	0.044*	
N2	0.4543 (3)	0.93097 (16)	0.83363 (11)	0.0398 (5)	
N3	0.6079 (3)	0.90671 (16)	0.79078 (12)	0.0408 (5)	
H3	0.623173	0.843446	0.770503	0.049*	
C1	−0.3546 (3)	1.06120 (19)	0.99967 (15)	0.0414 (6)	
H1A	−0.443263	1.076619	1.039357	0.050*	
H1B	−0.414913	1.065928	0.950101	0.050*	
C2	−0.2091 (3)	1.14674 (18)	1.00297 (12)	0.0347 (5)	
C3	−0.0077 (3)	1.00879 (18)	0.95616 (13)	0.0318 (5)	
C4	0.1523 (3)	0.98296 (18)	0.91927 (13)	0.0347 (5)	
H4	0.235910	1.037560	0.909001	0.042*	
C5	0.1897 (3)	0.87617 (17)	0.89736 (12)	0.0327 (5)	
C6	0.0621 (4)	0.79616 (19)	0.91380 (14)	0.0397 (6)	
H6	0.085751	0.724151	0.900581	0.048*	
C7	−0.1005 (4)	0.82224 (19)	0.94971 (14)	0.0413 (6)	
H7	−0.185698	0.768344	0.959433	0.050*	
C8	−0.1339 (3)	0.92836 (19)	0.97067 (13)	0.0350 (5)	
C9	0.3590 (3)	0.84945 (18)	0.85609 (12)	0.0334 (5)	
C10	0.4065 (4)	0.73195 (19)	0.84311 (15)	0.0502 (7)	
H10A	0.314605	0.686455	0.865158	0.075*	
H10B	0.414957	0.718030	0.788712	0.075*	
H10C	0.520253	0.716270	0.867180	0.075*	
C11	0.7324 (3)	0.98662 (18)	0.78224 (13)	0.0369 (5)	
C12	0.8963 (3)	0.95828 (19)	0.73614 (13)	0.0353 (5)	
C13	1.0105 (4)	1.0435 (2)	0.71613 (18)	0.0551 (7)	
H13	0.980509	1.114507	0.729847	0.066*	
C14	1.1677 (4)	1.0237 (3)	0.6761 (2)	0.0665 (9)	
H14	1.242362	1.081437	0.662510	0.080*	
C15	1.2150 (4)	0.9196 (3)	0.65617 (15)	0.0544 (7)	
H15	1.321087	0.906665	0.628974	0.065*	
C16	1.1054 (3)	0.8348 (2)	0.67653 (15)	0.0477 (7)	
H16	1.137637	0.763970	0.663540	0.057*	
C17	0.9464 (3)	0.85385 (19)	0.71639 (14)	0.0405 (6)	
H17	0.872727	0.795578	0.729944	0.049*	
O1W	0.4179 (3)	0.22231 (19)	0.82551 (14)	0.0727 (7)	

### N'-[(1E)-1-(3-Oxo-3,4-dihydro-2H-1,4-benzoxazin-6-yl)ethylidene]benzohydrazide monohydrate Atomic displacement parameters (Å^2^)

**Table:**

	*U*^11^	*U*^22^	*U*^33^	*U*^12^	*U*^13^	*U*^23^
O1	0.0415 (10)	0.0359 (9)	0.0657 (11)	−0.0009 (8)	0.0243 (9)	0.0007 (8)
O2	0.0432 (11)	0.0365 (9)	0.0609 (11)	0.0090 (8)	0.0028 (9)	−0.0107 (8)
O3	0.0499 (11)	0.0332 (9)	0.0655 (11)	0.0002 (9)	0.0116 (10)	−0.0081 (8)
N1	0.0282 (11)	0.0305 (10)	0.0525 (12)	−0.0007 (8)	0.0022 (9)	−0.0086 (9)
N2	0.0338 (11)	0.0396 (11)	0.0459 (11)	0.0059 (10)	0.0052 (10)	−0.0066 (9)
N3	0.0353 (11)	0.0340 (10)	0.0530 (12)	0.0025 (9)	0.0080 (10)	−0.0084 (9)
C1	0.0320 (12)	0.0417 (13)	0.0505 (14)	0.0008 (11)	0.0068 (12)	−0.0059 (12)
C2	0.0331 (12)	0.0365 (12)	0.0346 (11)	0.0048 (10)	0.0010 (11)	−0.0033 (10)
C3	0.0330 (13)	0.0270 (11)	0.0353 (12)	−0.0004 (10)	−0.0015 (10)	−0.0031 (10)
C4	0.0294 (12)	0.0310 (12)	0.0436 (12)	−0.0006 (10)	0.0015 (11)	−0.0026 (10)
C5	0.0346 (13)	0.0326 (11)	0.0309 (11)	0.0035 (10)	0.0005 (10)	0.0001 (9)
C6	0.0487 (15)	0.0281 (11)	0.0424 (13)	0.0019 (11)	0.0061 (13)	−0.0024 (10)
C7	0.0443 (15)	0.0320 (12)	0.0478 (14)	−0.0075 (12)	0.0078 (12)	0.0027 (10)
C8	0.0339 (13)	0.0364 (12)	0.0347 (11)	0.0008 (11)	0.0056 (10)	0.0005 (10)
C9	0.0359 (12)	0.0348 (12)	0.0294 (11)	0.0058 (11)	0.0008 (10)	−0.0014 (9)
C10	0.0559 (17)	0.0370 (14)	0.0577 (16)	0.0071 (13)	0.0198 (14)	0.0021 (12)
C11	0.0373 (13)	0.0322 (12)	0.0412 (12)	0.0016 (11)	0.0013 (11)	0.0020 (11)
C12	0.0336 (13)	0.0372 (13)	0.0350 (12)	0.0021 (11)	−0.0008 (10)	0.0020 (10)
C13	0.0529 (17)	0.0391 (15)	0.0731 (19)	−0.0037 (13)	0.0170 (16)	−0.0023 (14)
C14	0.0474 (18)	0.0582 (19)	0.094 (2)	−0.0145 (15)	0.0197 (17)	0.0046 (17)
C15	0.0351 (14)	0.073 (2)	0.0551 (16)	0.0064 (15)	0.0101 (13)	0.0020 (15)
C16	0.0444 (15)	0.0497 (15)	0.0492 (15)	0.0128 (14)	0.0014 (13)	−0.0043 (12)
C17	0.0373 (14)	0.0368 (13)	0.0476 (14)	0.0018 (12)	0.0029 (12)	0.0026 (11)
O1W	0.0842 (16)	0.0697 (15)	0.0643 (13)	0.0362 (14)	0.0257 (13)	0.0160 (12)

### N'-[(1E)-1-(3-Oxo-3,4-dihydro-2H-1,4-benzoxazin-6-yl)ethylidene]benzohydrazide monohydrate Geometric parameters (Å, º)

**Table:**

O1—C8	1.373 (3)	C6—H6	0.9300
O1—C1	1.427 (3)	C7—C8	1.375 (3)
O2—C2	1.235 (3)	C7—H7	0.9300
O3—C11	1.228 (3)	C9—C10	1.502 (3)
N1—C2	1.329 (3)	C10—H10A	0.9600
N1—C3	1.409 (3)	C10—H10B	0.9600
N1—H1	0.8600	C10—H10C	0.9600
N2—C9	1.285 (3)	C11—C12	1.495 (3)
N2—N3	1.391 (3)	C12—C17	1.378 (3)
N3—C11	1.355 (3)	C12—C13	1.390 (3)
N3—H3	0.8600	C13—C14	1.378 (4)
C1—C2	1.506 (3)	C13—H13	0.9300
C1—H1A	0.9700	C14—C15	1.369 (4)
C1—H1B	0.9700	C14—H14	0.9300
C3—C8	1.383 (3)	C15—C16	1.367 (4)
C3—C4	1.384 (3)	C15—H15	0.9300
C4—C5	1.393 (3)	C16—C17	1.385 (4)
C4—H4	0.9300	C16—H16	0.9300
C5—C6	1.393 (3)	C17—H17	0.9300
C5—C9	1.481 (3)	O1W—H1W	0.834 (13)
C6—C7	1.393 (4)	O1W—H2W	0.831 (13)
			
C8—O1—C1	115.12 (17)	O1—C8—C3	120.2 (2)
C2—N1—C3	122.4 (2)	C7—C8—C3	120.5 (2)
C2—N1—H1	118.8	N2—C9—C5	116.0 (2)
C3—N1—H1	118.8	N2—C9—C10	125.1 (2)
C9—N2—N3	116.36 (18)	C5—C9—C10	118.9 (2)
C11—N3—N2	117.42 (19)	C9—C10—H10A	109.5
C11—N3—H3	121.3	C9—C10—H10B	109.5
N2—N3—H3	121.3	H10A—C10—H10B	109.5
O1—C1—C2	113.9 (2)	C9—C10—H10C	109.5
O1—C1—H1A	108.8	H10A—C10—H10C	109.5
C2—C1—H1A	108.8	H10B—C10—H10C	109.5
O1—C1—H1B	108.8	O3—C11—N3	122.0 (2)
C2—C1—H1B	108.8	O3—C11—C12	121.6 (2)
H1A—C1—H1B	107.7	N3—C11—C12	116.3 (2)
O2—C2—N1	122.2 (2)	C17—C12—C13	118.4 (2)
O2—C2—C1	121.4 (2)	C17—C12—C11	124.6 (2)
N1—C2—C1	116.31 (19)	C13—C12—C11	116.9 (2)
C8—C3—C4	120.0 (2)	C14—C13—C12	120.4 (3)
C8—C3—N1	118.4 (2)	C14—C13—H13	119.8
C4—C3—N1	121.6 (2)	C12—C13—H13	119.8
C3—C4—C5	120.8 (2)	C15—C14—C13	120.6 (3)
C3—C4—H4	119.6	C15—C14—H14	119.7
C5—C4—H4	119.6	C13—C14—H14	119.7
C4—C5—C6	118.2 (2)	C16—C15—C14	119.5 (3)
C4—C5—C9	120.6 (2)	C16—C15—H15	120.2
C6—C5—C9	121.2 (2)	C14—C15—H15	120.2
C7—C6—C5	121.1 (2)	C15—C16—C17	120.4 (2)
C7—C6—H6	119.5	C15—C16—H16	119.8
C5—C6—H6	119.5	C17—C16—H16	119.8
C8—C7—C6	119.4 (2)	C12—C17—C16	120.6 (2)
C8—C7—H7	120.3	C12—C17—H17	119.7
C6—C7—H7	120.3	C16—C17—H17	119.7
O1—C8—C7	119.2 (2)	H1W—O1W—H2W	108 (4)
			
C9—N2—N3—C11	−164.5 (2)	N1—C3—C8—C7	−177.7 (2)
C8—O1—C1—C2	−43.3 (3)	N3—N2—C9—C5	−176.25 (18)
C3—N1—C2—O2	−177.5 (2)	N3—N2—C9—C10	3.4 (3)
C3—N1—C2—C1	0.2 (3)	C4—C5—C9—N2	−8.7 (3)
O1—C1—C2—O2	−153.8 (2)	C6—C5—C9—N2	169.9 (2)
O1—C1—C2—N1	28.4 (3)	C4—C5—C9—C10	171.7 (2)
C2—N1—C3—C8	−14.6 (3)	C6—C5—C9—C10	−9.7 (3)
C2—N1—C3—C4	166.7 (2)	N2—N3—C11—O3	2.1 (3)
C8—C3—C4—C5	−0.9 (3)	N2—N3—C11—C12	179.84 (18)
N1—C3—C4—C5	177.7 (2)	O3—C11—C12—C17	163.5 (2)
C3—C4—C5—C6	−0.2 (3)	N3—C11—C12—C17	−14.2 (3)
C3—C4—C5—C9	178.4 (2)	O3—C11—C12—C13	−12.5 (3)
C4—C5—C6—C7	1.3 (3)	N3—C11—C12—C13	169.7 (2)
C9—C5—C6—C7	−177.3 (2)	C17—C12—C13—C14	1.3 (4)
C5—C6—C7—C8	−1.3 (4)	C11—C12—C13—C14	177.6 (3)
C1—O1—C8—C7	−153.1 (2)	C12—C13—C14—C15	−0.7 (5)
C1—O1—C8—C3	30.4 (3)	C13—C14—C15—C16	−0.3 (5)
C6—C7—C8—O1	−176.3 (2)	C14—C15—C16—C17	0.6 (4)
C6—C7—C8—C3	0.1 (4)	C13—C12—C17—C16	−1.0 (4)
C4—C3—C8—O1	177.4 (2)	C11—C12—C17—C16	−176.9 (2)
N1—C3—C8—O1	−1.3 (3)	C15—C16—C17—C12	0.0 (4)
C4—C3—C8—C7	1.0 (3)		

### N'-[(1E)-1-(3-Oxo-3,4-dihydro-2H-1,4-benzoxazin-6-yl)ethylidene]benzohydrazide monohydrate Hydrogen-bond geometry (Å, º)

**Table:**

*D*—H···*A*	*D*—H	H···*A*	*D*···*A*	*D*—H···*A*
N1—H1···O2^i^	0.86	1.99	2.847 (3)	173
N3—H3···O1*W*^ii^	0.86	2.25	3.037 (3)	152
O1*W*—H1*W*···O2^iii^	0.83 (1)	2.10 (2)	2.925 (3)	169 (5)
O1*W*—H2*W*···O3^iv^	0.83 (1)	2.05 (2)	2.847 (3)	162 (3)
C10—H10*B*···O1*W*^ii^	0.96	2.34	3.200 (3)	150
C17—H17···O1*W*^ii^	0.93	2.52	3.228 (3)	133
C1—H1*B*···O3^v^	0.97	2.57	3.286 (3)	131
C16—H16···O3^vi^	0.93	2.58	3.447 (3)	155

Symmetry codes: (i) *x*+1/2, −*y*+5/2, −*z*+2; (ii) −*x*+1, *y*+1/2, −*z*+3/2; (iii) *x*+1/2, −*y*+3/2, −*z*+2; (iv) *x*, *y*−1, *z*; (v) *x*−1, *y*, *z*; (vi) −*x*+2, *y*−1/2, −*z*+3/2.
